# Towards an antimicrobial ‘microglove’

**DOI:** 10.1038/srep16679

**Published:** 2015-11-13

**Authors:** Ewoud Reilman, Joke G. Hagting, Theo Flipsen, Herb Ulmer, Jan Maarten van Dijl

**Affiliations:** 1 Department of Medical microbiology, University of Groningen, University Medical Center Groningen, Hanzeplein 1, P.O. Box 30001, 9700 RB Groningen, the Netherlands; 2 PolyVation B.V., Kadijk 7D, P.O. Box 70033, 9704 AA Groningen, the Netherlands

## Abstract

A large proportion of hospital-related infections are acquired and spread due to the direct contacts between patients and healthcare workers. Accordingly, proper infection prevention measures, and especially hand hygiene, are key to limit the spread of infections in nosocomial settings. However, healthcare workers frequently experience difficulties in complying strictly to hand disinfection protocols. This study was therefore aimed at the development of a hand rub with antimicrobial activity that forms a protective film on the hand, a so-called microglove, in order to enhance hand hygiene. For this purpose, various co-polymer formulations consisting of different ratios of Polyvinylpyrrolidone (PVP) and a branched C20 derivatized maleate (M20) in combination with the known biocide benzalkonium chloride (BKC) were tested for their combined film-forming and antimicrobial activities. The results of a series of novel contamination and transmission assays show that a formulation of 80% PVP and 20% M20 co-polymer with 0.9% BKC fulfils the elementary requirements for an antimicrobial microglove.

One of the major concerns for hospitals and other healthcare institutions is the continuous threat of infections caused by a wide range of opportunistic microorganisms. Hospitalized patients are especially susceptible for infections due to their underlying illnesses that may render them frail and/or immunocompromised. Additionally, wounds resulting from trauma or surgical interventions represent a breach of the primary skin barrier through which pathogens can readily gain access to normally well-protected body sites. Today, the majority of the infections caused by bacteria can still be treated reasonably well with antibiotics. However, the growing incidence of infections with antibiotic resistant bacteria makes treatment increasingly difficult and very costly^1^,^2^. This is underpinned by recent studies showing that infections with multi-drug resistant bacteria, such as methicillin resistant *Staphylococcus aureus* (MRSA), lead to increased morbidity, mortality and length of stay in hospitals^3^,^4^. Especially hospital-acquired (HA) bacterial infections are notorious for their drug resistant phenotypes, which originate from the continuously applied antibiotic pressure in nosocomial settings. Importantly, HA infections are to a large extent acquired and spread through direct contacts between patients and healthcare workers^5^,^6^,^7^. Therefore, a major prerequisite for success in the fight against HA infections is the strict implementation of effective infection prevention measures^8^.

One of the most effective precautions to minimize the spread of pathogens in healthcare settings is the routine decontamination of the hands of healthcare workers in-between patient contacts^7^,^9^. On a worldwide scale, it has been estimated that the strict implementation of standard control measures, in particular hand hygiene, could save one million lives annually^10^,^11^. Importantly, the success of the implementation of such control measures relies strongly on the strict adherence of healthcare workers to the protocol for hand disinfection between patient contacts. Yet, it has been noted that many healthcare workers experience difficulties in complying strictly to hand disinfection protocols for a range of different reasons 12,13,14. A key problem resides in the fact that effective hand hygiene requires the frequent re-application of soaps and alcohol- or chlorhexidine-based disinfectants. This can, on the long term, negatively affect the quality of the skin, resulting in skin irritation^15^. In addition, skin damage due to the use of aggressive hand-washing products makes the skin more prone to colonization by pathogenic micro-organisms^16^.

To circumvent the drawbacks of the repeated use of soaps or other disinfectants, the present study was aimed at the development of a new antimicrobial hand coating – a so-called ‘microglove’ - with a protective effect that would last in the minute to hour time range. Ideally, the antimicrobial microglove should consist of a thin comfortable polymer film that can be applied in the form of a hand rub, and that then covers the surface of the hands. Furthermore, to avoid interference with established hygiene regimens, the microglove coating should be readily removable by hand washing with regular soap and water. We therefore focused the present study on developing a Polyvinylpyrrolidone (PVP)-based hand rub, since PVP is a water-soluble excipient that is widely used both in the pharmaceutical industry, the food industry (E1201), and in numerous personal care products (17; http://www.fda.gov/default.htm). For example, PVP is applied in syrups, soft gelatin capsules, shampoos, toothpastes, hair sprays and gels, and in contact lens solutions. Accordingly, PVP is generally considered safe. In addition, its unique physical and mechanical properties make PVP an ideal candidate for a polymer-based hand rub. By using PVP in combination with a derivatized maleate it is possible to create PVP-based co-polymers, so called Aegimers, with different solubility properties^18^. In the present study, we therefore tested various co-polymer formulations consisting of different ratios of PVP and a branched C20 derivatized maleate named M20 for their film and texture properties. For proof-of-principle, the polymer formulations were combined with the known biocide benzalkonium chloride (BKC). BKC is a quaternary ammonium compound with chemical properties that make it interesting for use as a biocidal, cationic surfactant and phase transfer agent^19^. The antimicrobial activity of quaternary ammonium compounds, such as BKC, is primarily caused by their disorganizing effect on the plasma membrane^20^. As a biocidal, BKC has been applied in a large range of cosmetic products, non-alcoholic hand sanitizers, skin antiseptics, wet towels, mouth washes, and ophthalmic preparations, even though it can cause irritations of the skin^21^. The resulting polymer-biocide formulations were analyzed for their antibacterial activity. Using simulated contamination and transmission assays, a promising candidate ‘microglove’ formulation was identified, which consists of a co-polymer of 80% PVP and 20% M20 supplemented with 0.9% BKC (in short PVPM20-80:20-0.9% BKC).

## Results

### PVPM20 can function as coating-carrier for benzalkonium chloride

**Figure 1:**
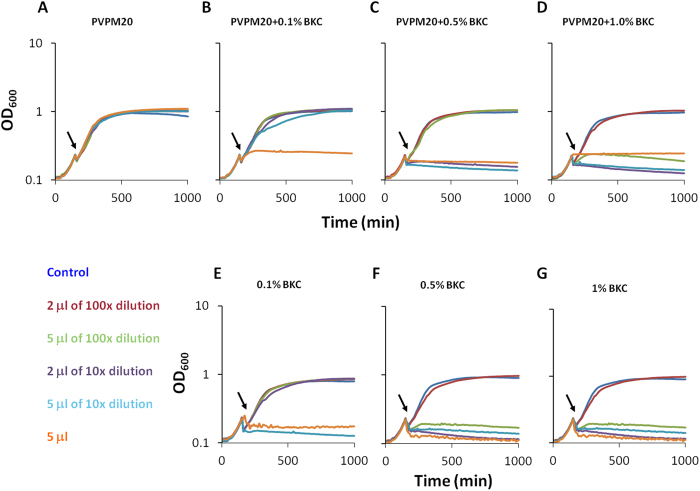
Growth of *S. aureus* HG001 in polymer-coated microtiter plates. Different amounts of serially diluted PVPM20-90:10 formulations and unaided BKC solutions were used to coat wells in a 96-well microtiter plate. These PVPM20-90:10 formulations and unaided BKC solutions contained increasing BKC concentrations as indicated. *S. aureus* HG001 was pre-cultured in 150 μl TSB using uncoated 96-well microtiter plates until early exponential growth after which aliquots of 100 μl were transferred to the polymer-coated wells. This transfer from one plate to another plate results in the observed drop in OD_600_ at the time point indicated by the arrows. Subsequently, growth at 37 °C for 1000 min was monitored by optical density readings at 600 nm. Notably, in experiment 1D, the killing efficiency of the undiluted formulation is apparently decreased compared to the 10-fold dilution. Although this is suggestive of a decreased killing activity, we consider it more likely that this observation relates to the formation of small aggregates by the respective polymer formulation, which would result in an increased OD_600_.

An antimicrobial microglove should be composed of a thin polymer film that is retained on the surface of the hand for a period of time in the minutes to hours range. In this study, such a polymer film was created through a co-polymer formulation consisting of PVP and M20 (i.e. PVPM20). In a first approach, PVP and M20 were used in a 90% to 10% ratio, respectively. Different PVPM20-90:10 formulations that either contained 0.1%, 0.5% or 1.0% BKC, or that lacked BKC, were tested for their antimicrobial activity against *S. aureus*, a major nosocomial pathogen with a high impact on morbidity, mortality and length of hospital stay^22^. To this end, aliquots of an exponentially growing *S. aureus* HG001 culture in Tryptic Soy Broth (TSB) were exposed to the different polymer coatings applied to the bottom of 96-well microtiter plate wells. The *S. aureus* HG001 strain was used for these analyses, because it is a well-characterized strain that is frequently used in studies on staphylococcal antibiotic resistance, infection and physiology^23^,^24^,^25^,^26^,^27^. Coatings with the PVPM20 polymer, but without BKC had no effect on growth of *S. aureus* HG001 at 37 °C as the cells that were introduced into the wells with only PVPM20-90:10 showed comparable growth rates as cells introduced into the untreated wells (Fig. 1A). This showed that PVPM20 itself has no antimicrobial activity. When we supplemented the PVPM20-90:10 with 0.1% BKC, the application of 5 μl of undiluted coating resulted in a complete inhibition of growth. However, when this formulation was diluted 10-fold, neither the 2 μl nor the 5 μl coatings were able to inhibit growth (Fig. 1B). By increasing the concentration of BKC to 0.5% the growth-inhibiting power of the polymer formulation increased considerably. In this case, coatings of 2 μl and 5 μl of the 10-fold diluted PVPM20-90:10 with 0.5% BKC were efficiently preventing growth of *S. aureus* (Fig. 1C). Growth inhibition was even further enhanced by using a PVPM20-90:10 formulation with 1.0% BKC, where even the 5 μl coating of a 100-fold diluted formulation was sufficient to stop the growth of *S. aureus* (Fig. 1D). Interestingly, coatings of BKC without the PVPM20 polymer, were slightly more effective in stopping the growth of *S. aureus* HG001 (Fig. 1E–G).

**Figure 2:**
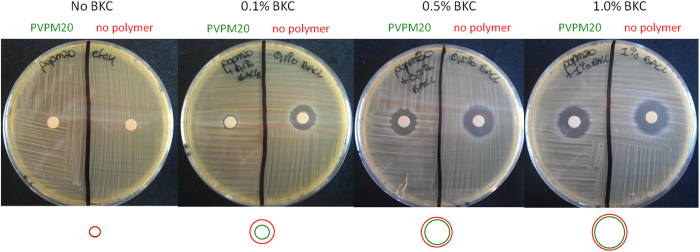
BKC disk diffusion assay in the presence or absence of PVPM20-90:10. TSA plates were inoculated confluently with *S. aureus* HG001. Next, Whatman paper disks loaded with BKC at different concentrations (0.1%, 0.5% or 1%) either with or without PVPM20-90:10 were placed on top of the plates. After overnight incubation at 37 °C growth inhibition zones were detectable around the paper disks. The circles underneath the images of the plates represent the respective sizes of the inhibition zones when disks were loaded with BKC alone (red) or with BKC plus PVPM20-90:10 (green); the red circle underneath the first plate (no BKC) indicates the size of the paper disk.

The observation that the PVPM20-90:10 polymer formulation slightly decreased the antibacterial effects of BKC suggested that the polymer coating of the microtiter plate inhibited the release of BKC into the culture medium. This idea was tested in a disk diffusion assay using Tryptic Soy Agar (TSA) plates confluently inoculated with *S. aureus* HG001. After overnight incubation at 37 °C, the growth inhibition zones around the paper disks were examined. Upon comparison of the growth inhibition zones around paper disks with PVPM20-90:10 *plus* BKC or with BKC alone, but both containing BKC at the same concentration, it was clearly evident that PVPM20-90:10 indeed inhibited the diffusion of BKC into the surrounding agar medium. Already at a BKC concentration of 0.1% the PVPM20-90:10 polymer resulted in a substantial reduction of the inhibition zone (Fig. 2). This inhibitory effect of PVPM20-90:10 on BKC diffusion became less prominent when higher concentrations of BKC were used. The latter observation can be explained by the higher concentration gradient of BKC in the PVPM20 relative to the surrounding agar medium, resulting in a faster release of the BKC and effectively more BKC that is available to diffuse from the paper disk into the surrounding agar medium. Alternatively, the PVPM20 coating may become saturated with BKC, allowing the BKC that is available in excess to diffuse rapidly from the paper disk into the surrounding agar medium. The fact that PVPM20-90:10 can set a limit to the diffusion of BKC into the surrounding medium implied that PVPM20 could represent an attractive slow-release carrier for antimicrobial compounds, such as BKC. In turn, this made the PVPM20-90:10 formulation with BKC an attractive candidate for further proof-of-principle studies on the antibacterial microglove concept.

### PVPM20-80:20-0.5% BKC effectively prevents *S. aureus* transmission

**Figure 3:**
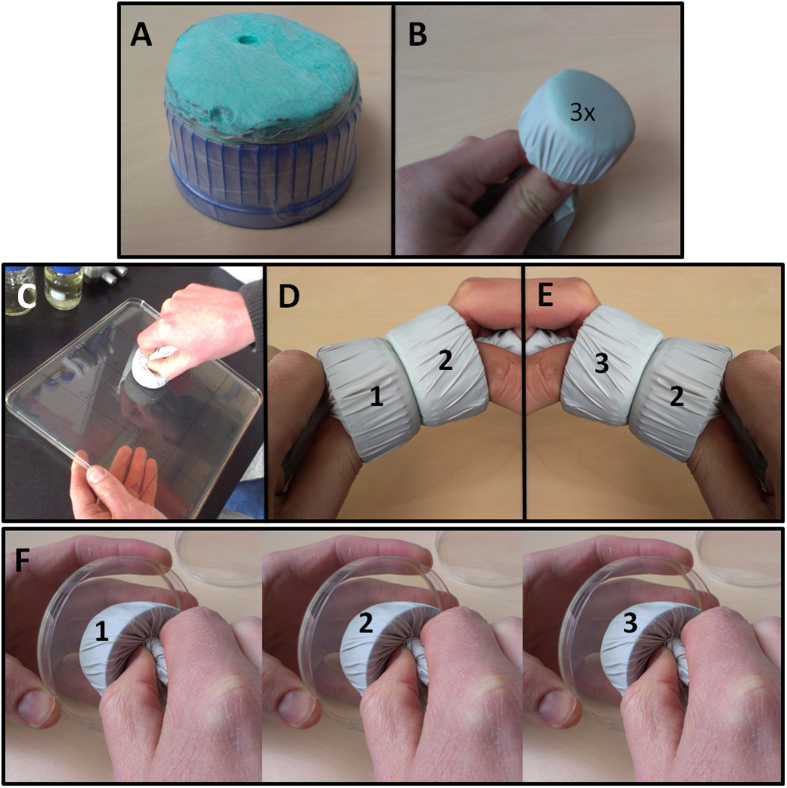
Design of a contamination and transmission assay. (**A**) Stamp design; stamps were made of screw caps for laboratory flasks on top of which absorption paper was fixed with parafilm. (**B**) Example of one of the three stamps wrapped with a nitrile examination glove. (**C**) Contamination procedure; a first stamp (no. 1) was pressed for 10 sec onto a TSA plate inoculated with *S. aureus* HG001. (**D**) First transmission step; stamp no. 1 was pressed to stamp no. 2 for 5 sec. (**E**) Second transmission step; stamp no. 2 was pressed to stamp no. 3 for 5 sec. (**F**) Contamination of stamps with *S. aureus* HG001 was assessed by pressing the stamps onto TSA plates.

**Figure 4:**
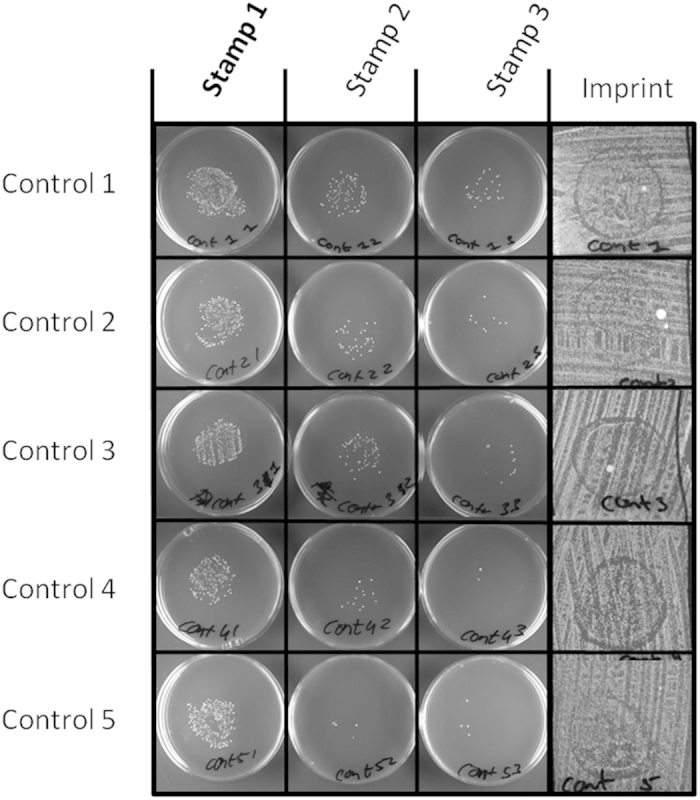
Controls for the contamination and transmission assay. The first three columns depict the contamination of the three stamps with *S. aureus* HG001 as reflected by colony formation on the fresh TSA plates onto which the non-polymer-coated stamps were pressed. The last column shows the imprint that was left on the ‘contamination plate’ (inoculated with *S. aureus* HG001) after overnight incubation.

**Figure 5:**
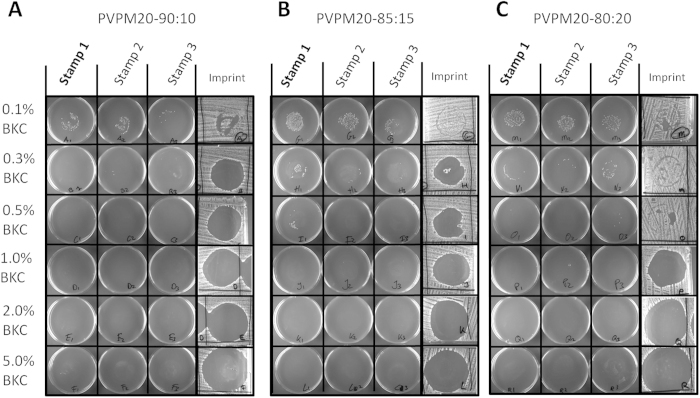
Effects of polymer coating on contamination and transmission. Stamps were coated with (**A**) PVPM20-90:10, (**B**) PVPM20-85:15, or (**C**) PVPM20-80:20 supplemented with different concentrations of BKC as indicated. The first 3 columns depict the contamination of the three stamps with *S. aureus* HG001 as reflected by colony formation on the fresh TSA plates onto which the stamps were pressed. The last column shows the imprint that was left on the contamination plate (inoculated with *S. aureus* HG001) after overnight incubation.

In the initial experiments described above, PVP and M20 were used in a 90% to 10% ratio. Since the PVP:M20 ratio is an important parameter for the properties of the co-polymer film that is to represent a microglove, we synthesized three formulations with different PVP:M20 ratios, namely 90:10 (PVPM20-90:10), 85:15 (PVPM20-85:15), and 80:20 (PVPM20-80:20). These co-polymer formulations were tested in combination with different BKC concentrations in an in-house developed contamination and transmission assay of which the different steps are schematically represented in Fig. 3. Briefly, stamps covered with a nitrile examination glove were either coated with a co-polymer formulation or they were left untreated. These stamps were contaminated by pressing them on a TSA plate inoculated with *S. aureus* HG001 (Fig. 3C). Next the stamp was pressed against a second stamp (Fig. 3D) and the second stamp was then pressed against a third stamp (Fig. 3E). All three stamps were subsequently pressed on fresh TSA plates (Fig. 3F), which were incubated overnight at 37 °C. This assay demonstrated that using the non-coated control stamps, transmission of *S. aureus* HG001 was detectable from the initially contaminated stamp to both the second and third stamps (Fig. 4). The numbers of transmitted bacteria decreased visibly after each transfer. Furthermore, the imprints left by the control stamps on the inoculated bioassay plate that was used for stamp contamination showed only the outline of the stamps and, as expected, there was no inhibition of bacterial growth. When stamps were coated with the original PVPM20-90:10 formulation containing 0.1% BKC (Fig. 5A), there was still a considerable amount of bacterial transmission by the three stamps. Contamination of the first stamp was slightly reduced compared to the uncoated control stamps. The imprint on the bioassay plate showed a small clearing zone, which suggests that part of the polymer coating was released upon contact with the agar. This can be explained by the fact that BKC residing in the polymer coating will diffuse into the agar, resulting in the clearing zones in which bacterial growth is inhibited. Increasing the concentration of BKC to 0.3% (Fig. 5A) prevented transmission to stamps no. 2 and no. 3, and even stamp no. 1 did not transmit viable bacteria, suggesting that the antibacterial coating was successfully applied. However, the imprint on the bioassay plate was characterized by a large clearing zone (Fig. 5A). This is likely due to the diffusion of BKC, which implies the release of some of the polymer film applied to the stamp. Increasing the BKC concentration to 0.5%, 1%, 2% and 5% resulted in even larger clearing zones (Fig. 5A). This can be explained as a direct consequence of increasing the BKC concentration. However, this can, at least in part, also be attributed to a secondary effect of the increasing BKC concentrations, since BKC is a well-known surfactant and phase-transfer catalyst that may change the water-resistant properties of the polymer film. Upon changing the PVP and M20 ratio to 85% and 15%, respectively (i.e. PVPM20-85:15), the addition of 0.1% BKC did not prevent the contamination of stamps no. 2 and no. 3 (Fig. 5B). Increasing the BKC concentration to 0.3%, which proved to be effective when using the PVPM20-90:10 formulation, was also not sufficient to fully prevent transmission to stamp no. 3. Nevertheless, the number of transmitted bacteria was clearly reduced. This was most likely due to some release of the polymer film, as was also reflected by the large clearing zone on the inoculated bioassay plate (Fig. 5B). Applying PVPM20-85:15 with a BKC concentration of 0.5% prevented bacterial transmission to stamp no. 2 and no. 3, but could not prevent contamination of the stamp no. 1. When the BKC concentration was increased to 1% and higher, none of the stamps showed contamination. However, the large clearing zones on the inoculated bioassay plate were indicative for the release of the polymer film obtained when PVP and M20 were present at a ratio of 80% and 20%, respectively (i.e. PVPM20-80:20). In combination with 0.1% BKC, the PVPM20-80:20 was still not sufficient in preventing transmission to stamp no. 3, However, the coating of the stamps with PVPM20-80:20 containing 0.3% BKC resulted in a substantial reduction of *S. aureus* HG001 transmission, although it was not completely prevented. Importantly, in this case, no clearing zones were detectable on the inoculated bioassay plate used to start the transmission experiment (Fig. 5C). Increasing the BKC concentration to 0.5% completely abolished the contamination of all three stamps, again without generating a clearing zone on the inoculated bioassay plate (Fig. 5C). This indicates that while the polymer film remained intact on the stamp it was able to prevent stamp contamination with bacteria and their subsequent transmission. PVPM20-80:20 co-polymer films containing BKC concentrations of 1% or more released substantial amounts of BKC onto the inoculated bioassay plate, as reflected by large clearing zones (Fig. 5C). Based on the significant antibacterial activity of the PVPM20-80:20 supplemented with 0.5% BKC, and on the stable film that it forms, the PVPM20-80:20 formulation was selected for further testing.

### PVPM20-80:20 coating facilitates slow BKC release

**Figure 6:**
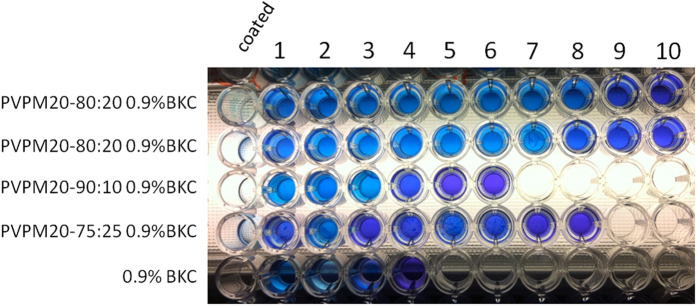
Release of BKC from different polymer formulations. Picture of the 96-well microtiter plate assay where the first well (marked ‘coated’) was used for the polymer coating and subsequent incubation steps with bromophenol blue (BPB) solution. Wells 1-10 contain the BPB solutions after incubation with the coating in the first well. The applied polymer formulations and the respective BKC concentrations are indicated. Of note, for unknown reasons, the BKC batch used for this experiment and the experiments in Fig. 7 had a slightly lower bactericidal activity than the batch used for the experiments shown in Figs 1, 2, 3, 4, 5. Therefore, the BKC concentration was increased to 0.9%. Furthermore, in case of the PVPM20-75:25 formulation, the structure of the film is compromised by the addition of water. As a result, small pieces of film are transferred to the other wells, which is clearly visible in well numbers 1, 2, 4, 5 and 6. These pieces of film still release some BKC.

The slow BKC release properties of the PVPM20-80:20 co-polymer formulation were verified in a BKC release test that was performed in a 96-well plate. In this experiment the PVPM20-90:10, PVPM20-80:20 and PVPM20-75:25 formulations were supplemented with 0.9% BKC and coated to the first well of each row of a 96-well plate. After evaporation of the 2-propanol solvent, an aqueous bromophenol blue (BPB) solution was added to the coated wells and the plate was incubated for 1 min at room temperature. BPB forms a blue complex with free BKC when it is released from the polymer film into aqueous solution. Of note, the BPB-BKC complex is blue, whereas the BPB solution itself is purple^28^. After incubation, the solution was transferred to the second well, and the color shift was assessed visually. Subsequently, new BPB solution was added to the polymer-BKC-coated wells, and incubated for 1 min and transferred to the third well. This course of actions was repeated until blue BPB-BKC complexes were no longer observed. The number of cycles needed to release readily detectable amounts of BKC was used as a measure to assess the BKC-retaining properties of the different polymer formulations. The results of this test showed that the PVPM20-80:20 formulation was most efficient in releasing BKC from the film (Fig. 6), as it took up to 8 consecutive incubations with the aqueous BPB solution until BKC was no longer detectably released (Fig. 6). In contrast, the film made with the PVPM20-75:25 formulation showed BKC release for only 2 incubations, after which the film completely disintegrated. The PVPM20-90:10 formulation performed slightly better, but after 5 consecutive incubations with the BPB solution no detectable amounts of BKC were released from the film. These findings were consistent with the results shown in Fig. 5, which indicated that the PVPM20-80:20 formulation was the most suitable candidate formulation for further testing. Additionally, we tested the BKC release from a 0.9% BKC coating without PVPM20-80:20. In this case, all coated material dissolved instantaneously upon addition of the BPB solution and all BKC was thereby released. Of note, the BKC concentration in commercially available formulations is lower (~0.2%) than the concentration used in our present polymer formulations. However, the BKC-release assay shows that by using BKC in combination with the PVPM20-80:20 polymer, the actual release of free BKC from the polymer film is considerably lower than that of unaided BKC as implemented in commercial BKC-based disinfectants. Combined with the other data presented above, it can thus be concluded that the high concentration of BKC is retained within the PVPM20-80:20 polymer film and is only slowly released upon contact with water. This slow BKC release combined with the good film properties implied that the PVPM20-80:20 0.9%BKC formulation matched the basic requirements for a hand rub that facilitates the establishment of an antibacterial microglove.

### Validation of the antibacterial microglove concept

**Figure 7:**
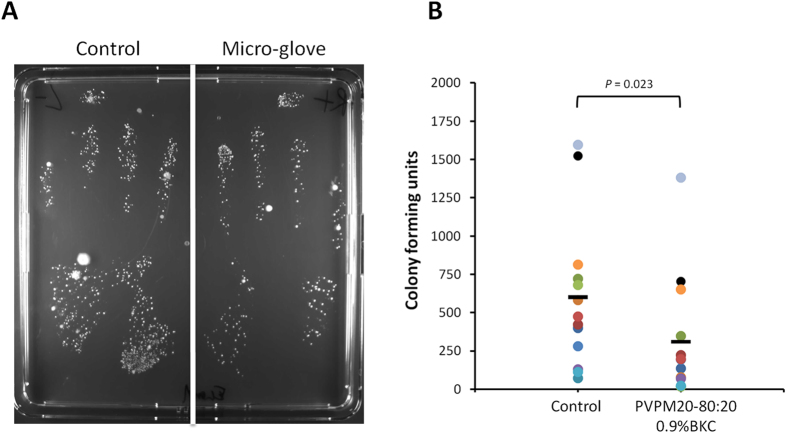
Effects of polymer coating on microbial glove contamination. (**A**) Example plate depicting the contamination of polymer-coated and non-coated examination gloves. The gloves were worn by a volunteer for 3 hours during which time the volunteer performed regular activities. Subsequently, the volunteer gently pressed the gloves onto a bioassay plate with LB agar. The picture was taken with the Syngene G:box after overnight incubation at 37 °C. In this example, the left glove was used as an uncoated control, while the right glove was coated with PVPM20-80:20 0.9% BKC. (**B**) Results of the glove contamination assays. Coated and non-coated gloves were worn by 13 volunteers for about 3 hours. Colony forming units on the LB plates onto which the used gloves were pressed, were counted with the Syngene software. The outcome of each individual experiment is indicated with a different color code, allowing the comparison of the contamination of each pair of coated and non-coated gloves. The uncertainty in counting was marginal as this was done automatically, giving always the same outcome as long as the camera settings were not altered. In this respect it is relevant to note that the CFU’s obtained for the control gloves were always determined at the same time as the CFU’s obtained from the coated gloves. Statistical analyses were done using the Mann–Whitney U test. Horizontal black bars indicate average numbers of colony forming units.

To test whether the PVPM20-80:20 0.9%BKC formulation could function as a microglove that offers protection against microbial contamination, its functionality was evaluated using a glove contamination assay. In this assay 13 volunteers were asked to wear a PVPM20-80:20 0.9%BKC-treated and an untreated examination glove (control), while performing their normal daily activities with the exception of hand washing and disinfection. After approximately 3 hours, both gloved hands were pressed gently on a Lysogeny Broth (LB) agar plate to assess the levels of microbial glove contamination. The plates were incubated overnight at 37 °C, and the next day the colony-forming units (CFUs) on the plates (Fig. 7A) were counted. Indeed, the gloves coated with the PVPM20-80:20 0.9%BKC formulation yielded significantly lower numbers of CFUs on the inner hand surface than the non-coated gloves, demonstrating a protective antimicrobial effect of the polymer coating (Fig. 7B). Overall, the numbers of CFUs were approximately halved when a glove was treated with PVPM20-80:20 0.9%BKC, but in some individual experiments the effect was substantially more prominent with up to 40-fold reductions in CFUs. For unknown reasons, other experiments showed less pronounced antimicrobial effects of the polymer coating. Even so, in each single experiment the coating of a glove with PVPM20-80:20 0.9%BKC led to a reduced number of microbial contaminants compared to the respective control (Fig. 7B). This shows that the microglove concept has indeed a considerable protective effect against newly acquired contaminants for a period of approximately 3 hours.

## Discussion

In this study, we provide the proof-of-principle for a new type of antimicrobial hand rub that forms a protective microglove and has the potential to be used as an alternative for the current disinfectants applied by healthcare workers. The great advantage of the antimicrobial microglove concept is that it reduces the risks of microbial hand contamination for healthcare workers and, consequently, the risks of transmission of pathogens from healthcare workers to patients for a period of time that is sufficiently long for patient examination and the provision of care. Due to its antimicrobial activity, the microglove may reduce the critical need for repetitive hand disinfection procedures. Importantly, since in principle fewer hand disinfection events are needed for protection, the microglove could potentially enhance the compliance of healthcare workers with established hygiene protocols.

The present microglove formulation is based on a co-polymer of PVP and the branched C20 derivatized maleate M20. This co-polymer is dissolved at a concentration of 5% in 2-propanol and is supplemented with 0.9% BKC. Like in commercially available alcohol-based disinfectants, such as Sterillium^®^, the initial disinfection is most likely caused by the solvent 2-propanol. However, after the 2-propanol evaporates, a thin polymer film containing the active biocide BKC remains on the skin forming the protective microglove. Of note, there are other BKC-based disinfectants currently on the market, but these do not claim to protect users against renewed microbial contamination after they have disinfected their hands.

Most of the currently commercially available BKC-based products contain about 0.2% BKC, while the present microglove formulation contains 0.9% BKC. Importantly, our present findings indicate that not all of this BKC is instantaneously released from the co-polymer film. Instead, the BKC is retained by the polymers and slowly released into the environment. We consider this as an advantageous property to protect hands against microbial contamination, and also to limit the subsequent transmission of microbial contaminants. Furthermore, the fact that most of the BKC in the microglove formulation remains confined in the polymer film, and is not immediately released, is likely to limit the actual hand exposure to BKC, thereby minimizing a possible irritation of the skin by BKC.

In our present study, optimal BKC release was observed for the PVPM20-80:20 co-polymer formulation. The rate of BKC release is mainly determined by the hydrophilicity of the co-polymer film. The hydrophilicity determines the degree of swelling in aqueous media and, with that, the permeability for the water-soluble BKC. In this respect, the PVPM20-90:10 co-polymer film is more hydrophilic and takes up more water resulting in faster release of BKC compared to the PVPM20-80:20 co-polymer film. The film of PVPM20-80:20 and the film of PVPM20-90:10 adhere both strongly to the bottom of the surface-modified polar polystyrene well. The less hydrophilic and thus more apolar film of PVPM20-75:25 adheres less effectively to the bottom of the well. Consequently, during transfer of the BPB medium, pieces of the PVPM20-75:25 film are transferred to the second well, which disturbs the imaging. In other words, the BKC release from coated films also depends on adhesion phenomena.

In conclusion, the present study demonstrates the feasibility of a disinfecting hand sanitizer that can be regarded as an antimicrobial microglove. The microglove formulation that was tested provided increased protection against newly acquired microbial contaminants for a period of at least three hours. Although our microglove concept is technically feasible, it is key to realize that this type of product cannot replace strict hygiene protocols. Instead, it should be regarded as a tool that is complementary to existing hygiene protocols, and that can potentially enhance the efficacy of such protocols. Lastly, it should be noted that further optimization studies will be needed before an antimicrobial microglove can be implemented in the daily routine. In the first place, it would be important to further increase the bactericidal activity of the microglove to enhance the protective effect. Furthermore, the current microglove formulation and/or the amounts of polymer applied convey a certain stickiness that could be perceived as unpleasant. However, a great advantage of the PVPM20 co-polymer system is that its chemical and physical properties can be easily modified and this, in combination with optimization of the hand rub formulation, provides ample possibilities for further development towards a novel solution in reducing hospital-acquired infections.

## Methods

### Strains and growth media

*S. aureus* HG001^23^ was grown in TSB or on TSA. Liquid *S. aureus* HG001 cultures were grown in 96-well plates at 37 °C and under constant agitation using a Biotek powerwave microplate reader.

### Co-polymers

Co-polymers with different ratios of PVP and M20 were prepared by PolyVation BV. These different co-polymers (in short PVPM20) were dissolved to a final concentration of 5% in 2-propanol. The resulting PVPM20 solutions were then supplemented with different concentrations of BKC (Sigma Aldrich).

### Co-polymer screening for antimicrobial activity

The co-polymer antimicrobial activity screen was performed with PVP and M20, mixed at a 90% to 10% ratio, respectively, and supplemented with 0.1%, 0.5% or 1.0% BKC from a 50% (w/v) stock solution. PVPM20-90:10 without BKC, and BKC solutions of 0.1%, 0.5% or 1.0% without PVPM20 were used as controls. The different formulations were used to coat 96-well microtiter plates by applying different aliquots to the bottoms of the wells; 5 μl of the original 5% PVPM20 solutions, 2 μl and 5 μl of 0.5% PVPM20 solutions (10x diluted), and 2 μl and 5 μl of 0.05% PVPM20 solutions (100x diluted). The wells were air-dried, resulting in the deposition of a polymer film on the bottom of the wells. The BKC controls were applied using the same approach. Next, 100 μl aliquots of a culture of exponentially growing *S. aureus* HG001 in TSB were added to the wells and growth was monitored for 14 hours by optical density readings at 600 nm (OD_600_) using a Biotek powerwave plate reader at maximal shaking.

### Disk diffusion assay

PVPM20-90:10 (5%) formulations with either 0.1%, 0.5% or 1% BKC were spotted in 5 μl aliquots on 5 mm Whatman^®^ paper disks. Alternatively, 5 μl aliquots of 0.1%, 0.5% or 1% BKC solutions were spotted on the disks. After disk drying at room temperature, the disks were placed on TSA plates onto which *S. aureus* HG001 had been spread to obtain a confluent lawn of cells. These plates were then incubated overnight at 37 °C and, the next day, the sizes of the observed inhibition zones were measured to estimate the diffusion of BKC from the paper disks

### *S. aureus* contamination and transmission assay

To assay the impact of different polymer formulations on the contamination of surfaces with *S. aureus* and the subsequent *S. aureus* transmission to other surfaces, a dedicated assay was developed. Briefly, an overnight culture of *S. aureus* HG001 was diluted 1:10.000 and 1 ml was plated confluently onto two large bioassay plates with TSA. After inoculation, the plates were dried at 37 °C for approximately 30 min to allow bacteria to settle and to remove access moisture. Nitrile examination gloves were wrapped around self-fabricated stamps (Fig. 3, A and B), which were made from absorption towel placed on a bottle cap and secured by a parafilm wrapping (Fig. 3A). The gloved stamps were coated with 50 μl polymer formulations, or they were left untreated (control). Contamination and transmission was achieved by pressing the gloved stamp (no. 1) onto the plate inoculated with *S. aureus* for approximately 10 sec (Fig. 3C). The stamp was then used to contaminate a second stamp (no. 2) by pressing the two together for 5 sec (Fig. 3D), after which it was pressed for 5 sec onto a clean TSA plate (Fig. 3F). Subsequently, the second stamp was first pressed against a third stamp (no. 3; Fig. 3E), and both stamps were then pressed onto clean TSA plates for 5 sec (Fig. 3F). All plates were incubated overnight at 37 °C. Bacterial growth on the agar plates, including that on the two bio-assay plates used for the initial contamination of stamp no. 1, was used to assess the quality and anti-bacterial capacity of the polymer films applied to the stamps. Importantly, we included five non-coated control stamps, which were pressed onto different locations on the bioassay plates, to preclude a possible position-related assay bias.

### BKC release assay

The different polymer formulations (i.e. PVPM20-90:10, PVPM20-80:20, and PVPM20-75:25 in 2-propanol) were supplemented with 0.9% BKC and 20 μl coated to the first well of each row of a 96-well plate. After evaporation of the 2-propanol solvent, 100 μl of an aqueous BPB solution (6 × 10^−4^ mmol/L) was added to the coated wells and the plate was incubated for 1 min at room temperature. Next, the aqueous phase was removed from the well and the formation of blue BPB-BKC complexes was assessed by visual inspection. This process was repeated until blue BPB-BKC complexes were no longer observed, and the number of repeated BPB incubation steps was recorded.

### Glove contamination assay

Nitrile examination gloves (Sterling Nitrile Powder-Free Exam Gloves, Kimberly-Clark) were coated with 1 ml of PVPM20-80:20 (5%) dissolved in 2-propanol and supplemented with 0.9% BKC. As a control, untreated nitrile examination gloves were used. Next, 13 volunteers were asked to wear a PVPM20-80:20 0.9%BKC-treated and an untreated examination glove (control), while performing their normal daily activities. To prevent a dominant hand bias, the coated glove was randomly assigned to the left or right hands of the volunteers. After ~3 hours, both gloved hands were pressed gently on a LB agar plate which was then incubated overnight at 37 °C. Images were recorded with a G:BOX gel documentation and analysis system (Syngene). The numbers of CFUs on the plate were automatically assigned using the Syngene software package. CFU numbers thus determined were used as a measure for the numbers of microbial contaminants that had adhered to the glove.

## Additional Information

**How to cite this article**: Reilman, E. *et al.* Towards an antimicrobial ‘microglove’. *Sci. Rep.* **5**, 16679; doi: 10.1038/srep16679 (2015).
